# Metabolic profiles identify circulating biomarkers associated with heart failure in young single ventricle patients

**DOI:** 10.1007/s11306-021-01846-8

**Published:** 2021-10-03

**Authors:** Thomas M. O’Connell, David L. Logsdon, Gloria Mitscher, R. Mark Payne

**Affiliations:** 1 Department of Otolaryngology-Head & Neck Surgery, Indiana University School of Medicine, 1300 W. Michigan St, Suite 400, Indianapolis, IN 46202 USA; 2 Department of Anatomy, Cell Biology & Physiology, Indiana University School of Medicine, Indianapolis, IN USA; 3 Indiana Center for Musculoskeletal Health, Indiana University School of Medicine, Indianapolis, IN USA; 4 Division of Cardiology, and Herman B Wells Center for Pediatric Research, Department of Pediatrics, Indiana University School of Medicine, Indianapolis, IN USA

**Keywords:** Single ventricle, Metabolomics, Heart failure, Biomarker, Children

## Abstract

**Background:**

Children and young adults with single ventricle (SV) heart disease frequently develop heart failure (HF) that is intractable and difficult to treat. Our understanding of the molecular and biochemical reasons underlying this is imperfect. Thus, there is an urgent need for biomarkers that predict outcome and provide a rational basis for treatment, and advance our understanding of the basis of HF.

**Objective:**

We sought to determine if a metabolomic approach would provide biochemical signatures of HF in SV children and young adults. If significant, these analytes might serve as biomarkers to predict outcome and inform on the biological mechanism(s) of HF.

**Methods:**

We applied a multi-platform metabolomics approach composed of mass spectrometry (MS) and nuclear magnetic resonance (NMR) which yielded 495 and 26 metabolite measurements respectively. The plasma samples came from a cross-sectional set of young SV subjects, ages 2–19 years with ten control (Con) subjects and 16 SV subjects. Of the SV subjects, nine were diagnosed as congestive HF (SVHF), and 7 were not in HF. Metabolomic data were correlated with clinical status to determine if there was a signature associated with HF.

**Results:**

There were no differences in age, height, weight or sex between the 3 cohorts. However, statistical analysis of the metabolomic profiles using ANOVA revealed 44 metabolites with significant differences between cohorts including 41 profiled by MS and 3 by NMR. These metabolites included acylcarnitines, amino acids, and bile acids, which distinguished Con from all SV subjects. Furthermore, metabolite profiles could distinguish between SV and SVHF subjects.

**Conclusion:**

These are the first data to demonstrate a clear metabolomic signature associated with HF in children and young adults with SV. Larger studies are warranted to determine if these findings are predictive of progression to HF in time to provide intervention.

**Supplementary information:**

The online version contains supplementary material available at 10.1007/s11306-021-01846-8.

## Introduction

The origins of HF in children and young adults are strikingly different from those in adults and most often involve structural abnormalities in heart formation (Hsu & Pearson, 2009). The SV heart is a particularly severe form of congenital heart disease and results from underdevelopment of either the right ventricle, or the left ventricle, such as with hypoplastic left heart syndrome (HLHS) (Ohye et al., 2016). The SV is ultimately palliated with a surgery called the Fontan procedure, which uses systemic venous pressure to drive blood flow through the lungs leaving the SV to pump to the body (Fredenburg et al., 2011).

This innovative surgical and medical management has allowed children with SV hearts to live into early adulthood, but long-term management remains a significant clinical problem for adults with congenital heart disease. Most children do well in the first decade of life, but their hearts begin to fail in the 2nd and 3rd decade and the outcome is frequently death or crippling HF as teenagers or young adults (d'Udekem et al., 2014). In the pediatric heart network longitudinal study of Fontan patients at 21.4 years of age, 10% of the patients had received a heart transplant for HF, and 5.6% had died (Atz et al., 2017). The incidence of HF among young adults with congenital heart disease (CHD) is 1.2 per 1000 patient-years, and one-year mortality for patients with CHD after hospital admission for HF is 24% (Zomer et al., 2013). Freedom from adverse events is approximately 50% at 16 years for the SV Fontan surgical repair (d'Udekem et al., 2014).

Heart failure in the adult population is an acquired disease and is overwhelmingly due to coronary artery insufficiency and subsequent ischemia of the heart. In contrast to adult HF however, coronary blood supply is rarely compromised in children and young adults with congenital heart disease. Here, failure of the SV is not due to ischemia but rather, from abnormal hemodynamic conditions leading to molecular and cellular events that have yet to be determined. In addition, it is increasingly clear that the long-term impact of the Fontan circuit results in a congestive hepatopathy leading to liver dysfunction, fibrosis, cirrhosis, and rarely hepatocellular carcinoma (Emamaullee et al., 2020). The chronic elevation in central venous pressure from the Fontan operation also injures the bowel and is frequently associated with protein losing enteropathy (Atz et al., 2017; Pundi et al., 2015). Recent studies have begun to look for biomarker associations predictive of outcome with some success, such as NT-proBNP, but there remain few management guidelines, few predictive early biomarkers, and no animal models of SV leading to a poor understanding of the complications from SVHF today (van den Bosch et al., 2021). Therapeutic options are limited for SVHF and the ability to predict outcome or track improvement with therapeutic interventions are also limited (Emamaullee et al., 2020). Indeed, most new concepts for diagnosis and management of HF in children today are based on translation of adult HF treatment strategies with little preclinical evidence supporting their use in the young. As a result, clinical management of failure of the SV has not advanced significantly in the past 2 decades, and the field lacks an understanding of the molecular events and biomarkers that predict failure and outcome (Reddy et al., 2020)**.**

There is a significant body of literature where metabolomics methods have been applied to adult HF (reviewed in McGarrah et al., 2018) In contrast, there is only a very limited application of this technology to adult patients with SV, and none in children and young adults with SV. We find only two papers describing metabolomic investigations focused on patients with SV (Michel et al., 2020a, 2020b). Both of these studies analyzed the same cohort of 20 older adult Fontan patients with age/sex matched controls using mass-spectrometry-based metabolomics. The SV patients were reported to have significant alterations in amino acid, phospholipid, and acylcarnitine metabolism.

In the current study we applied a multi-platform metabolomics analysis to plasma from a cohort of healthy controls (Con, n = 10), SV patients (n = 7), and SVHF (n = 9). We hypothesized that we would be able to discern a more detailed metabolic phenotype of SV patients at a younger age using a more comprehensive metabolomics approach, and that we could find unique signatures for the SVHF patients. Our goal was to better understand the potential metabolic derangements in SV and SVHF, and search for biomarkers that may be used to identify SV patients at risk for progression to HF.

These results are the first to show metabolic derangements in children and young adults with SV, and more importantly, identify metabolomic profiles associated with HF in this group. This has not been done before now. Expansion of these findings will allow greater understanding of the basic mechanisms underlying HF and allow development of metabolomic profiles for management, and risk stratification of HF in children and young adults.

## Methods

### Study cohort

This study was reviewed and approved by the Indiana University School of Medicine Institutional Review Board and conformed to the declaration of Helsinki. All subjects gave written informed consent if older than 18 year, or assent if younger than 18 year (with parental consent), to participate in the study. From December 2018 to November 2019, children and young adults with SV were recruited from the pediatric cardiology clinics and inpatient cardiology service at Riley Hospital for Children, at Indiana University School of Medicine, Indianapolis, IN, USA. These subjects all had single ventricle hearts of either left ventricle, or right ventricle dominant anatomy, and all had undergone total cavopulmonary anastomosis. Of the 16 single ventricle subjects, 69% (11) self-identified as Caucasian race, 19% (3) as Black race, and 12% (2) as Hispanic race. Children and young adults without heart disease served as controls (Con) and were recruited from the pediatric cardiology clinics. Inclusion criteria for Con subjects included structurally normal hearts. The Con subjects were all healthy and did not have any systemic disease or other heart disease. Of the 10 Con subjects, 100% (10) self-identified as Caucasian race. Management of subjects with SV was based on the standard of care (SOC), and the diagnosis of heart failure was based on clinical judgement of the attending cardiologist for that subject. Exclusion criteria for both cohorts were the absence of any other metabolic or systemic diseases, such as diabetes, cystic fibrosis, inflammation or infection, or cancer, and must not be pregnant.

Clinical data was abstracted from the medical records after consent was obtained. Subjects were not fasted overnight prior to blood draw. Between 5 and 10 ml of venous blood was drawn into EDTA anticoagulant tubes from subjects in a sitting position. Bloods were immediately spun at room temperature (20 °C) for 10 min at 2500 × g to separate cellular blood elements from plasma, and the plasma was aliquoted and stored at − 80 °C until analysis. Samples were transported on dry ice for analyses.

### NMR-based metabolomics

Samples for NMR analyses were prepared using established protocols (Beckonert et al., 2007)**.** Briefly, 100 μl of serum was combined with 300 μl of deuterated phosphate buffered saline (PBS) and the solution was filtered through a 10KDa molecular weight cutoff filter. The filtrate was then combined with deuterated PBS containing contain 2,2-dimethyl-2-silapentane-5-sulfonate-d6 (DSS-d6) to a final volume of 600 ul and 0.5 mM DSS-d_6._ Spectra were acquired on a Bruker AVANCE III, 700 MHz NMR equipped with a cryogenically-cooled probe. Data was acquired using the 1D NOESY pulse sequence covering 12 ppm, digitized with 32,768 points during a 3.9 s acquisition time. The mixing time was set to 100 ms and the relaxation delay between scans was set to 2.0 s. The data was processed and quantitative spectral fitting of 26 metabolites was carried out using the Chenomx NMR Processor and Profilers software packages (Chenomx Inc., Edmonton, Alberta, Canada) with DSS-d6 as the chemical shift and quantitation standard.

The NMR platform was used to profile some of the higher concentration metabolites not included in the targeted MS platform. Examples include organic acids such as formate, citrate, and succinate as well as carbohydrates including glucose and glycerol. Some metabolites, such as amino acids were detected by both platforms. When detected by both platforms, the MS derived value was used.

### Targeted MS-based metabolomics

The targeted MS experiments used the Biocrates Q500 kit run on an AB Sciex 5500 QTRAP with an Agilent 1290 UPLC (Biocrates AG. Innsbruck, Austria). This is a commercially available targeted metabolomics assay that can be used on a variety of LC/MS/MS instruments. Preparation of serum samples followed vendor protocols. In brief, 10 µL of the supplied internal standard solution were added to each well (except for the zero sample) on a filterspot of the 96-well extraction plate. After drying under a gentle stream of nitrogen 10 µL of each serum sample, quality control (QC) samples, blank, zero sample, or calibration standard were added to the appropriate wells. This assay targeted 629 metabolites across a wide range of chemical classes including amino acids and amino acid related compounds, bile acids, free fatty acids, phosphatidylcholines, sphingomyelins, ceramides, cholesterol esters, diglycerides and triglycerides. Isotope labeled standards for a subset of these metabolites are included in this assay to support quantitation. A subset of 134 metabolite had more than 50% zeros and were therefore not included in the analysis and thus, at total of 495 metabolites were included in the analyses As set of eight QC samples were prepared from pooled sample and included in the run. Data processing was carried out using the Biocrates MetIDQ software. The results of both the NMR and MS platforms were reported in micromolar units. Supplementary Table 1 contains all of the metabolites.

### Data integration and biomarker analysis

Integration, statistical analysis and visualization of the metabolomics data was carried out using the VIIME platform (www.viime.org) (Choudhury et al., 2020) along with custom scripts written in the R programming language. Statistical analyses to detect significantly altered metabolites were carried out using ANOVA with Tukey post-hoc testing. Given the small size of the data, further false-discovery rate analyses were not conducted.

The large dynamic range of the metabolite measurements dictated that we apply a log transformation and Pareto scaling to both datasets during integration. Heatmaps were generated in viime using z-scores and the K-nearest neighbor hierarchical clustering algorithm. The receiver operator characteristic (ROC) analysis was carried out in MetaboAnalyst (version 5.0) using the multivariate exploratory analysis functions. Briefly, ROC curves were generated by Monte-Carlo cross-validation with classification and ranking carried out by Partial Least Squares Discriminant Analysis (PLS-DA) using two latent variables.

## Results

Twenty-six patients provided written consent and were enrolled in the study. Subjects were not fasted for this study. Ten patients had no heart disease and were enrolled in the Con group. Sixteen patients had a diagnosis of SV of which 8 had a diagnosis of HLHS, and 8 had a diagnosis of other forms of SV including double inlet left ventricle, tricuspid atresia, unbalanced atrioventricular canal, and double outlet right ventricle. Of those 16 patients with SV, 9 were diagnosed with congestive HF based on physical examination and required diuretic support. This cohort was designated as SVHF. Seven single ventricle patients did not have a diagnosis of HF, were not on diuretic support, and were designated as SV. The baseline characteristics for each of the three groups are shown in Table 1.

**Table 1:** Patient baseline characteristics

	Control (n = 10)		SV (n = 7)	SVHF (n = 9)	*p value*	
Epidemiological background						
Age (years)	9.16 (2.2–14.3)	11.01 (7.5–15.3)		8.82 (2.8–19.7)		0.64
Sex (% male)	3 (30%)	5 (71%)		3 (33%)		0.20
Height (cm)	137.1 (90.5–171)	141.6 (122–176.3)		119.8 (93.7–158.5)		0.18
Weight (kg)	40.15 (16.9–72.6)	41.07 (24.7–87.3)		29.8 (13.3–88.5)		0.52
BMI (kg/m2)	19.75 (14.2–29.1)	19.41 (15.2–28.1)		18.69 (14.1–35.2)		0.91
Diagnosis				Normal heart		
HLHS			3 (43%)	5 (56%)	0.64	
DILV/TA/AVC/DORV			4 (57%)	4 (44%)	1.0	
Surgical procedure(s)						
Fontan extra-cardiac			0 (0%)	3 (33%)	0.10	
Fontan lateral tunnel			7 (100%)	6 (67%)	0.10	
Age at Fontan (months)			28.3 (11–57)	36.44 (18–44)	0.29	
Medications						
β-blocker			1 (14%)	3 (33%)	0.42	
ACE-inhibitor/ARB			4 (57%)	7 (78%)	0.41	
Digoxin			1 (14%)	4 (44%)	0.22	
Diuretic			0 (0%)	9 (100%)	< 0.0001	
Aspirin			5 (71%)	7 (78%)	0.79	

Multi-group comparisons by one-way Anova. Two-group comparisons by unpaired t test with the exception of Diuretic use (Mann–Whitney test). Continuous measurements are reported as mean and range (parenthesis), whereas categorical measurements are reported as total and percentage (parenthesis). Unadjusted *p* values in right hand column with significance set at the 0.05 level

*AVC* unbalanced atrioventricular canal, *DILV* double inlet left ventricle, *DORV* double outlet right ventricle, *HLHS* hypoplastic left heart syndrome, *TA* tricuspid atresia

There were no significant differences in age, sex, height, or weight between all 3 groups. In addition, there were no significant differences in the anatomic diagnosis, age at surgery, or type of surgical palliation between the SV and SVHF cohorts. The mean oxygen saturation was not different between SV groups (92.9% vs. 92.1%). Cardiac troponin I (cTnI) and C-reactive protein (CRP) were measured in 5 of 7 SV subjects, and 8 of 9 SVHF subjects. The CRP was normal in all subjects except for one child (2.2 years) at 2.9 mg/dL, and the cTnI was normal at < 0.03 ng/mL in all subjects measured. Medication use between the cohorts was not different except for diuretic use in the SVHF group (p < 0.0001). Within the SVHF cohort, 3 had protein losing enteropathy (PLE), and 3 required heart transplantation because of severe HF at a later date. In contrast, no SV patient was being treated with diuretics, had PLE, or required heart transplantation.

### Metabolomics analysis yields distinct metabolic phenotypes of SV and SVHF

**Fig. 1:**
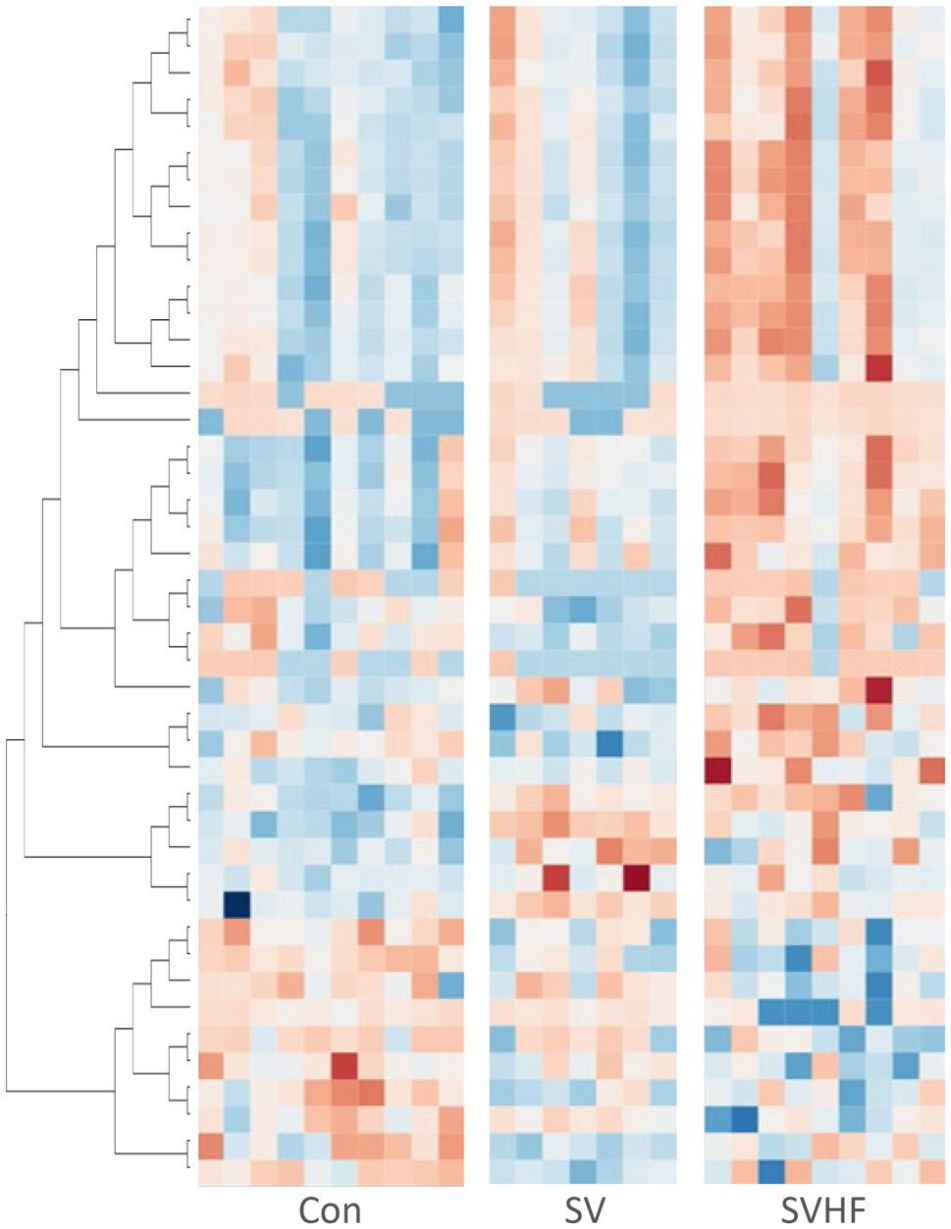
Heatmap of 44 metabolites for which there was a significant difference in at least one of the inter-group comparisons. The z-scores of the metabolites were used in the preparation of the heatmap with blue color indicates lower and red indicates higher metabolites concentrations. Metabolites were ordered using K-nearest neighbor hierarchical cluster analysis as implemented in viime

**Fig. 2:**
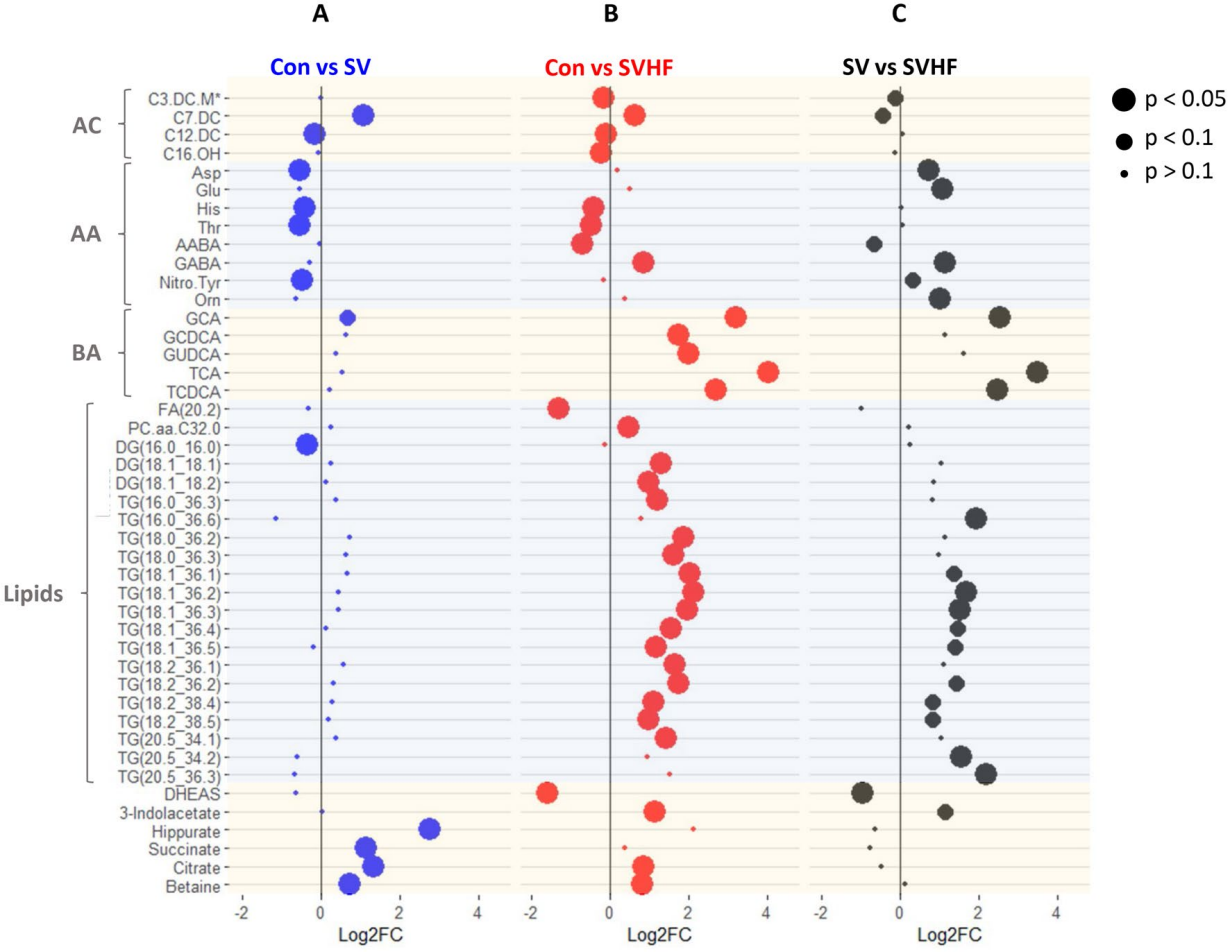
Forest Plot of individual metabolite changes between groups with the log2-fold change plotted on the X-axis. **A** Control versus single ventricle. **B** Control versus single ventricle heart failure and **C** Single ventricle versus single ventricle heart failure. Dot size indicates statistical significance: large dots = p < 0.05, medium dots = p < 0.1, and small dots = p > 0.1. *Note that C3.DC.M is isobaric with the other short chain AC, hydroxyvalerylcarnitine (C5.0H) and is an alternative assignment

Integration of the NMR and MS data revealed a set of 44 metabolites for which there was a significant difference in at least one of the inter-group comparisons. Of these significant metabolites, 41 were detected by MS and 3 were detected by NMR. Supplementary Table 1 contains the ANOVA derived p-values for all of the metabolites detected by both platforms. When a metabolite was detected by both platforms, only the MS was listed. A heatmap of the significantly altered metabolites is shown in Fig. 1. This figure was generated using a hierarchical analysis of the metabolites and clearly shows a difference in the metabolite patterns for the three groups. To further detail the metabolic differences between the three groups, the individual metabolites changes are represented as forest plots in Fig. 2. A comparison of Con versus SV patients is shown in Fig. 2A with the log2-fold change for each metabolite shown. Figure 2B and C present similar comparisons of Con versus SVHF, and SV versus SVHF respectively.

A set of 4 acylcarnitines (ACs) were found to be significantly altered, including dicarboxylated (DC) and hydroxylated (OH) species. The short chain, methylmalonylcarnitine (C3-DC-M) was slightly reduced in SVHF compared to both controls and SV. The medium chain pimeloylcarnitine (C7.DC) was increased in both SV and SVHF compared with controls. Trends toward reduced levels of these short and medium chain ACs were observed in SVHF versus SV. The longer chain dodecanedioylcarnitine (C12-DC) was reduced in both SV and SVHF, and the hydroxyhexadecanoylcarnitine (C16-OH) was significantly reduced only in the SVHF group. For these longer chain ACs no significant difference was observed between SV and SVHF cohorts.

A set of 8 amino acid-related compounds were significantly altered. Of these, four were canonical amino acids: aspartate (Asp), glutamate (Glu), histidine (His) and threonine (Thr). The significant changes included reductions in both SV and SVHF compared with controls. The SVHF patients were characterized by increased levels of Asp and Glu compared with SV.

The four non-standard amino acids include nitro-tyrosine, ornithine, α-aminobutyric acid (AABA) and γ-aminobutyric acid (GABA). AABA was significantly reduced in SVHF and trended downward in comparing SVHF with SV. In contrast, GABA was significantly increased in SVHF compared to both Con and SV. A significant reduction in nitro-tyrosine is only observed in the Con versus SV comparison along with a trending reduction in SVHF compared with SV. Ornithine demonstrated a significant increase in SVHF compared with Con.

Detailed analysis of bile acids in young Fontan patients has not been reported before now, nor related to HF status. A set of five bile acids were found to be significantly altered including the secondary bile acid glycocholic acid (GCA) and the conjugated primary bile acids glycochenodeoxycholic acid (GCDCA), glycoursodeoxycholic acid (GUDCA), taurocholic acid (TCA) and taurochenodeoxycholic acid (TCDCA). All alterations involve increases with a trending increase in GCA in the SV group while four of the five are significantly elevated in the SVHF group. Strikingly, three bile acids, TCA, GCA, and TCDCA, showed significant increases which clearly distinguished the SVHF patients from the SV cohort.

Alterations in lipids are represented by changes in 21 metabolites with 16 of these being triacylglycerols (TGs). Interestingly, there were no changes in the TGs in the SV group when compared with the Con cohort, but 13 of these were significantly increased in the SVHF group compared with Con. A set of 5 significant increases in TGs were observed in the SVHF group compared with SV, along with trending increases in 6 others.

Additional metabolic changes were found which suggest potential effects on hormone signaling, energy metabolism, and one-carbon metabolism. These have not been described before now in the child with SV. A significant decrease in the androstane steroid, dehydroepiandrosterone sulfate (DHEAS) was observed between Con versus SVHF which persisted when comparing SV versus SVHF. Additional changes were observed in 3-indoleacetate and hippurate. The Krebs cycle intermediates citrate and succinate were both increased in Con versus SV, whereas only the increase in citrate was observed in the Con versus SVHF. Neither Krebs cycle intermediate was different between SV and SVHF. Betaine functions mainly as a methyl donor that plays a significant role in a number of processes including liver function. Significant increases in betaine were observed in the Con versus SV, and Con versus SVHF comparisons.

### Diagnostic capabilities of the metabolite profiles

**Fig. 3:**
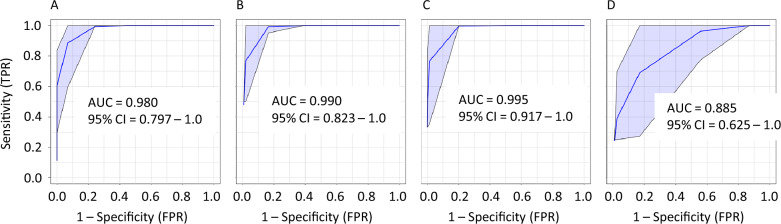
Receiver operating characteristic analyses of metabolite profiles. ROC curves were generated using PLS-DA classification and feature ranking methods to distinguish (**A**) Con from all SV subjects including both SV and SVHF (**B**) Con from SV (**C**) Con from SVHF and (**D**) SV from SVHF. Blue shaded regions indicate the 95% confidence intervals. The metabolites included in these analyses are those shown in Fig. 2

To evaluate the potential of the metabolite profiles as a diagnostic panel, receiver operating characteristic curves were generated using partial least squares discriminant analysis models. Figure 3 shows the area under the curve (AUC) values along with the 95% confidence intervals (CI) for four comparisons. Figure 3A evaluates the ability to discriminate between the control patients and the combined SV and SVHF group. Figure 3B shows the comparison between the control and SV group. The AUC and CI for this model are slightly better than for the combined group further suggesting that the SV and SVHF models are indeed metabolically distinct. The best performing ROC analysis was between the control and SVHF with the largest AUC value and the narrowest CI; Fig. 3C. As expected, the AUC and CI comparing SV and SVHF (Fig. 3D) are weaker than the comparisons with controls, but these values still show high discrimination between SV and SVHF cohorts, and support the concept that the metabolite panel has significant potential to discriminate SV patients from those with HF.

## Discussion

**Fig. 4:**
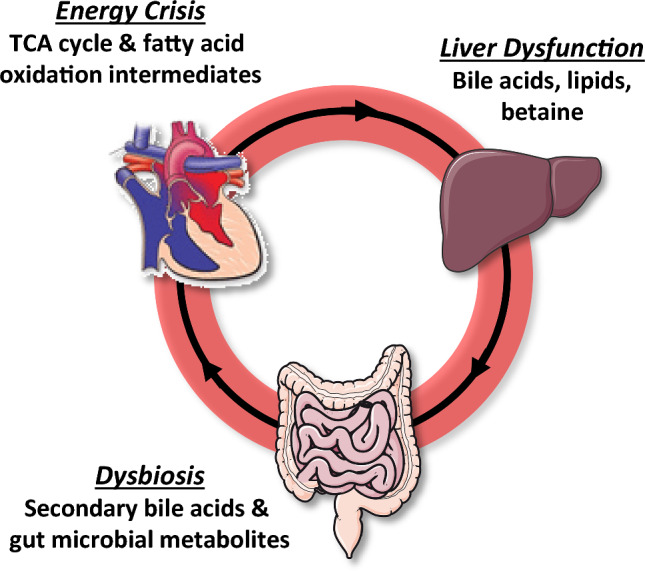
Systemic derangements in SV patients lead to distinct metabolic patterns of heart failure. A heart with a hypoplastic left ventricle is shown after an intermediate stage repair (Glenn shunt) prior to the Fontan operation. Here, the poor functioning of the heart can negatively affect downstream organs, such as liver and bowel. This altered physiology may, in turn, negatively affect cardiac function and the metabolites released by these organs may constitute a heart failure profile

The metabolic profiles found in this pilot study significantly expand upon the limited metabolomic studies of SV subjects. Furthermore, these findings demonstrate that: (1) specific metabolic profiles are associated with HF in these patients, (2) these profiles can distinguish between CON, SV, and SVHF and, (3) are found in early childhood. Multiple studies evaluating HF in the SV subject to this point have relied on clinical laboratory data, such as BNP, or multiplex immunoassays, and have advanced our understanding of inflammation and tissue responses at a protein level for the Fontan subject (van den Bosch et al., 2021). However, the biochemical profiles in this pilot study provide new clues regarding the metabolic pathways that are altered in SV, and which patients may progress to HF. Figure 4 provides an overview of how the various organs may be affected by the SV heart physiology, and how these metabolites may signal specific tissue dysfunction. This information is critically lacking in the field of congenital heart disease and will allow both clinical and basic interrogation of the Fontan physiology.

The results of the recent studies by Michel et al., invites a detailed comparison to the results presented here (Michel et al., 2020a, 2020b). Similar, albeit more limited, targeted metabolomics approaches were used in those studies and their cohort was similar in size to ours. Some critical differences are found in the subjects included in their study when compared to those of the current study. In the Michel studies, all of the SV subjects had a dominant left ventricle with 50% of the subjects having a double inlet left ventricle, and 50% having a variation of tricuspid atresia. In our study, 50% of SV subjects had HLHS and thus, had dominant right ventricular anatomy. As has been noted by others, the outcome is not the same between right and left dominant SV hearts with significantly better function and survival of the LV dominant hearts (Erikssen et al., 2018; Oster et al., 2018). Secondly, subjects with failure of the systemic ventricle were excluded from the Michel studies whereas ours included them. Indeed, three of the subjects in our study went on to transplant due to intractable HF. Our study also included subjects with protein losing enteropathy (PLE) whereas those subjects were excluded in the Michel study. Finally, our study focused on children and young adults (age range 2.2–19.7 years) whereas the Michel study included only adults and excluded subjects < 18 year of age. The results from Michel et al. demonstrated that the metabolome in adult SV patients can be distinguished from controls. The results from our study progress this distinction further and demonstrate that the metabolic phenotype of pediatric SV patients with heart failure is indeed distinguishable from pediatric SV patients with stable heart function.

Perturbations in the acylcarnitine (AC) profiles were found in the study of adult SV patients by Michel et al. In that study, significant elevations in C0 (carnitine), C3 (propionylcarnitine) and C18:2 (octadecadienylcarnitine) were found in the SV patients. In contrast, we found perturbations only in dicarboxylated and hydroxylated species. Moreover, three of the four species were decreased while only one was increased; see Fig. 2.

Perturbations to ACs and specifically the dicarboxylated ACs, have been found in several studies of cardiovascular disease. In a metabolomics analysis of serum from 314 individuals with CAD, a principal components analysis derived metabolite factor composed of small to medium chain dicarboxylated ACs significantly predicted an incidence of myocardial infarction (MI) and death (Shah et al., 2010). In a similar analysis from the CATHGEN cohort, a factor composed of both short and long chain dicarboxylated ACs was independently associated with mortality and MI (Hunter et al., 2016). In our study, the C12-DC and C16-OH metabolites were the only longer chain AC with a significant perturbation, but only in comparing the controls to SV and SVHF and not in the comparison of SV to SVHF. The exact nature of changes in ACs in our study as well as the studies of adults with CVD is not clear, but these changes suggest that some perturbations to fatty acid metabolism in SV induced heart failure may be common with adult heart disease, while others may be distinct.

Amino acids are important as biosynthetic substrates for cellular structures and signaling molecules as well as being a source of energy. The circulating levels of amino acids are achieved in part by maintaining a balance between protein synthesis and degradation along with amino acid catabolism. Consistent with the study from Michel et al. we find decreases in Asp, His and Thr in the SV patients, and an increase in Glu in the SVHF compared with SV (Michel et al., 2020a, 2020b). Understanding the detailed nature of cellular energetics and amino acid biosynthetic and catabolic processes at play in the SV patient will require more study.

The alterations in BAs are an intriguing finding that likely relate to the congestive hepatopathy associated with the Fontan circuit (Emamaullee et al., 2020). Increases in serum BAs have been observed in a wide variety of liver diseases (Neale et al., 1971) but not specifically described in the Fontan SV setting before now. Interestingly, all five of the BAs were increased in the SVHF compared with controls and three of these were different between SVHF and SV suggesting they may be an early marker of hepatic pathology due to failure of the Fontan physiology.

Bile acid metabolism is also affected by gut microbiota and is known to alter metabolism of the host (Wahlstrom et al., 2016). The significant increase in the secondary BA, GCA in the SVHF group compared to both controls and SV raises the possibility that the gut microbiome may contribute to, or signal, HF in the SV patient. Further support for the role of gut microbiota is the increase in 3-indoleacetate, which is often produced as a product of gut microbial tryptophan metabolism. The role of the microbiota in the SV subject has not been investigated and may provide a new target to understand HF in these patients.

A significant pattern of increased triglycerides was observed in the SV and SVHF group suggesting that dyslipidemia may be a factor in SV heart failure. Michel et al. reported alterations in phospholipid metabolism as evidenced by reduced levels of phosphocholines and sphingomyelins. The increase in triglycerides found in our study may be associated with the alteration in lipolytic processes that can play a significant role in the development of adult heart failures (Kintscher et al., 2020).

Whitesides et al. reported significant reductions in circulating cholesterol including reductions in total cholesterol, low-density lipoprotein cholesterol and high-density lipoprotein cholesterol in a cohort of pediatric SV patients who underwent Fontan palliation (Whiteside et al., 2013). In a subsequent study they found evidence that the hypocholesterolemia was associated more with increased absorption rather than decreased synthesis (Whiteside et al., 2016). Their observation of elevated markers of liver dysfunction suggested that the hypocholesterolemia was related to the abnormal hepatic cholesterol metabolism. It is not clear how the increased levels of TGs in our study related to the decreased levels of cholesterol levels. As both cholesterol and triglycerides are components of the low-density lipoprotein particles, a more detailed examination of lipoprotein particle number and size could be revealing.

### Study limitations

Although our current study provides new insights into the metabolic pathophysiology of HF in the child with SV heart disease, there are several limitations. First, this is considered a small study consisting of 10 controls and 16 SV subjects. Single ventricle heart disease is rare and occurs in about 5 of every 10,000 live births and thus, recruiting pediatric subjects with a range of disease states is expected to be difficult (Hoffman & Kaplan, 2002; Reller et al., 2008). None-the-less, even with this cohort size our study shows highly significant differences between controls and subjects, and most excitingly, between SV and SVHF. When compared with the existing literature on biomarker analysis in the SV patient, however, our study is unique for its age range and the spectrum of disease state and has generated valuable insights into HF in the SV that will generate new mechanistic hypotheses. It is likely that a larger study will provide greater definition of the SV metabolome based on age and anatomy. Secondly, the age range in this study is large: 2 to ~ 19 years. Thus, a larger cross-sectional study would be needed to define how the metabolome changes with age as the child grows and/or HF ensues. Certainly, a larger longitudinal study will need to be designed to define analytes that may be predictive of outcome. Finally, HF in children is difficult to quantify and define as opposed to adults where rating scales, such as the New York Heart Association classification, can be used to categorize the limitations to physical activity (Hsu & Pearson, 2009). Small children, for example, cannot perform exercise testing. Other HF scales for children have been devised, such as the Ross scale (Ross, 2001), and the New York University Pediatric HF index (Connolly et al., 2001). Of these, the pediatric heart failure index correlates well with EKG, echocardiographic, and biochemical markers of HF better than the Ross or NYHA scoring systems (Tissieres et al., 2006), but none of these have been validated as surrogate clinical endpoints for children. In this study, the diagnosis of HF was based on clinical judgement as guided by feeding, growth rate, and physical examination, and treatment with diuretics which is standard of care in this age range (Ross, 2001). This definition is subject to observer bias and difficult to quantify. Larger, prospective clinical trials using classifications such as the pediatric heart failure index are needed to confirm and validate the findings in this current study.

## Conclusions

The expanded analyte profiles described here in children with SV hearts are novel and will allow further, hypothesis-driven studies into the metabolic perturbations of the SV patient. Furthermore, there are very clear metabolite differences between SV and SVHF subjects, which may allow for development of these analytes as biomarkers predictive of progression to HF, as well as response to therapeutic intervention. Future studies will need to involve cross-disciplinary expertise and techniques to understand organ dysfunction in the unique physiology of the SV Fontan heart.

## Supplementary information

Below is the link to the electronic supplementary material.

Supplementary file1 (XLSX 49 kb)
